# Editorial: Unhealthy language: linguistic investigations of COVID-19 discourse

**DOI:** 10.3389/frai.2023.1281059

**Published:** 2023-09-18

**Authors:** Justyna A. Robinson, Roberta Piazza, Rodney Jones

**Affiliations:** ^1^School of Media, Arts, and Humanities, University of Sussex, Brighton, United Kingdom; ^2^Department of English Language and Applied Linguistics, University of Reading, Reading, United Kingdom

**Keywords:** COVID-19, pandemic, text, language, discourse, communication

*Unhealthy Language: Linguistic investigations of COVID-19 discourse* aims to bring some clarity to a period of great disruption and chaos and the way, in the midst of this chaos, language – emanating from official sources and exchanged in our everyday lives – functioned to inform us, to scare us, to reassure us, and to help us make sense of the radical change we were going through. The original objective was to produce an agile, accessible, and scholarly reliable book that would follow a rapidly changing and volatile situation. Some of the papers therefore are the result of studies still in progress or just concluded. In other words, we tried to capture the immediacy of the ‘unprecedented' (COVID-19 buzz word) situation while it was still developing.

The choice of an open access volume also aligns with our determination to make the investigations of the book immediately accessible to everyone who wants to reflect on the COVID-19 phenomenon. The volume offers geographical, disciplinary and methodological diversity. It contains eight papers, written by 27 authors, from 14 universities/institutions, across six European countries (UK, Belgium, Ukraine, Bulgaria, Italy, Estonia). Several of the papers have are the result of a collaboration between linguists and health scientists. Data for the studies include official documents, public signs, media texts, interviews and diaries, and these data are approached from range of perspectives, i.e., computational, sociolinguistic, semantic-pragmatic, discourse analytical, and ethnographic.

One of the main challenges people faced during the pandemic was adapting to new health-related practices and regulations, some of which involved the development of new terminology and new genres of discourse and interaction. This is the focus of the paper by Bafort et al. based on research conducted in collaboration with the Flemish Agency of Health, entitled “*COVID-19 telephone contact tracing in Flanders as a “contested” new genre of conversation: discrepancies between interactional practice and media image*”. The authors analyse the interactional dynamics of contract tracing calls and compare their findings to media representation of such calls. They discover that the mainstream media's representation of contact tracing, which focused mostly on privacy concerns and the background of the tracers rather than the purpose and the conduct of the calls, presented a distorted image that may have had considerable consequences for the efficacy of contact tracing.

Another paper which highlights the mismatch between media representations and official discourses associated with the language of the pandemic is Kania “*Snake flu”, “killer bug”, and “Chinese virus”*: *A corpus-assisted discourse analysis of lexical choices in early UK press coverage of the COVID-19 pandemic*, in which she reveals how, contrary to WHO guidelines, UK newspapers regularly used terms such as “killer bug” and “Chinese virus” to refer to the virus, likely stoking fear and promoting racism among their readers.

Giorgis et al. “*We are at War” The Military Rhetoric of COVID-19 in Cross-cultural Discourses* focus on the discourse of both mainstream media and political speeches in Italy, Bulgaria, and Ukraine. The Authors examine how the metaphor of WAR, which is found in such constructions as “We are at war” or “We will win this war,” was used differently in different political and cultural contexts.

While the three papers described above focus on media discourse, others address similar issues in the discourse of ordinary people. Wilding et al. for example, in their paper “*A metaphor analysis of older adults' lived experience of household isolation during COVID-19*”, examine the way adults in the UK used metaphors to describe their experiences of lockdown. The Authors show how the participants negotiated their sense of agency by resisting and refashioning the dominant public metaphors that circulated as part of Government campaigns.

While Wilding et al. focus on how people coped through repurposing metaphors, Robinson et al. in their paper “*Introducing the keyconcept approach to the analysis of language: The case of REGULATION in COVID-19 diaries*” show how broader concepts were repurposed in the COVID-19 discourse of ordinary people. Focusing on how participants in the 12th May Diary project, which is part of the Mass Observation Archive, discursively constructed the keyconcept of REGULATION during the first COVID-19 lockdown in the UK, they show how the concept of REGULATION was associated with a complex collection of thoughts, feelings and experiences including the experience of limited individual agency and feelings of both fear and gratitude.

In another study which explores diary data curated during the first COVID-19 lockdown in the UK, Cowie et al. in their paper entitled “*Imagining the city in lockdown : Place in the COVID-19 self-recordings of the Lothian Diary Project*”, analyse audio and video diaries from residents of Edinburgh In particular they focus on how diarists made sense of disruptions in place-time during COVID-19 pandemic using three different narrative orientations or “chronotopes”.

The ways the pandemic disrupted people's experience of space is particularly evident in the papers that explore changes to the linguistic landscapes of European cities and towns during the pandemic. Bagna and Bellinzona in their paper “*Everything will be all right (?)”: discourses on COVID-19 in the Italian linguistic landscape*”, show how the interaction between public and private discourse in the linguistic landscape of Florence during different phases of the pandemic provides a window onto the ways citizens communicated about the “shared shock” of the pandemic and formulated social discourses and emotional responses to it.

Similarly, Tragel and Pikksaar in their paper “*Authority and solidarity on the Estonian COVID-19 signs*: *In line with the government's guidelines, we ask you to wear a mask*” explore the linguistic strategies used on door signs in Estonian cities and towns during the pandemic. The Authors identify the linguistic strategies people used to negotiate relationships of authority and/or solidarity between the authors of the signs and their readers.

The Research Topic includes a commentary on all eight papers by Jones entitled “*How to have agency in a pandemic*” in which he identifies *agency* as a key theme running through all of the papers and delineates how, in the range of contexts represented in these papers, people employed discourse as a tool to make sense of and, in some cases, challenge, constraints on their ability to take action, make choices, and control what was happening around them.

As these brief summaries suggests, these contributions capture people's attempts to cope with the new reality through formulating new ways of speaking, writing, acting and interacting and through adapting to or contesting the new discursive regimes that were imposed on them. What characterizes this volume is a linguistic focus accompanied by a deep interest in understanding how human nature can be resourceful and confront the unexpected. The book shows us how language functions as a socio-cognitive tool that people use both to make sense of reality, and to construct it.

## Author contributions

JR: Writing—original draft. RP: Writing—original draft. RJ: Writing—original draft.

